# Transposable Element Dynamics and Regulation during Zygotic Genome Activation in Mammalian Embryos and Embryonic Stem Cell Model Systems

**DOI:** 10.1155/2021/1624669

**Published:** 2021-10-15

**Authors:** Yixuan Low, Dennis Eng Kiat Tan, Zhenhua Hu, Shawn Ying Xuan Tan, Wee-Wei Tee

**Affiliations:** ^1^Chromatin Dynamics and Disease Epigenetics Group, Institute of Molecular and Cell Biology, Agency for Science, Technology, And Research (A^∗^STAR), Singapore 138673; ^2^Department of Physiology, Yong Loo Lin School of Medicine, National University of Singapore, Singapore 117593

## Abstract

Transposable elements (TEs) are mobile genetic sequences capable of duplicating and reintegrating at new regions within the genome. A growing body of evidence has demonstrated that these elements play important roles in host genome evolution, despite being traditionally viewed as parasitic elements. To prevent ectopic activation of TE transposition and transcription, they are epigenetically silenced in most somatic tissues. Intriguingly, a specific class of TEs—retrotransposons—is transiently expressed at discrete phases during mammalian development and has been linked to the establishment of totipotency during zygotic genome activation (ZGA). While mechanisms controlling TE regulation in somatic tissues have been extensively studied, the significance underlying the unique transcriptional reactivation of retrotransposons during ZGA is only beginning to be uncovered. In this review, we summarize the expression dynamics of key retrotransposons during ZGA, focusing on findings from in vivo totipotent embryos and in vitro totipotent-like embryonic stem cells (ESCs). We then dissect the functions of retrotransposons and discuss how their transcriptional activities are finetuned during early stages of mammalian development.

## 1. Introduction

**Figure 1:**
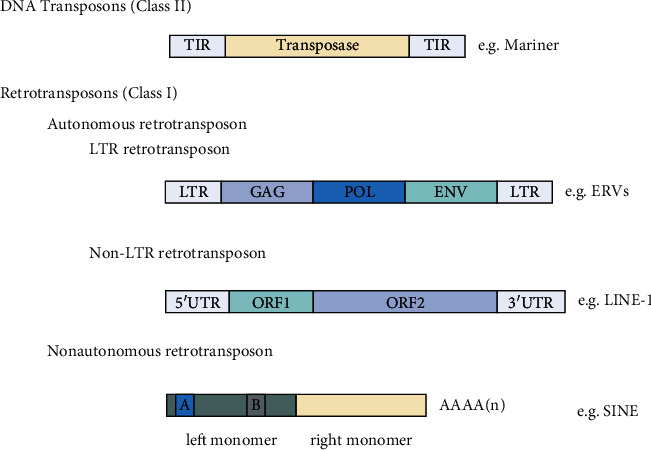
Schematic representations of key TE classes present in mammalian genomes. TEs can be broadly classified into DNA transposons (class II) and retrotransposons (class I). For a specific breakdown of each order, please refer to the review by [6]. Abbreviations: GAG: capsid protein; ENV: envelope protein; POL: coding region that encodes one or more of the following: proteinase, reverse transcriptase, RNaseH, polymerase, or integrase; TIR: terminal inverted repeat; LTR: long-terminal repeat; UTR: untranslated regions; EN: endonuclease; RT: reverse transcriptase; A and B: RNA polymerase III promoter box.

Annotations of eukaryotic genomes have revealed that repetitive elements interspersed between protein-coding genes are prevalent [1–3], constituting up to two-thirds of the human genome [4]. TEs are DNA sequences that can reintegrate to other genomic regions within the same cell of origin. Based on their mechanism of transposition, TEs can be divided into two main classes: retrotransposons (class I) and DNA transposons (class II) (Figure 1) [1, 5]. DNA transposons are the smallest class of mobile genetic elements, making up approximately 3% of the human genome, and they copy themselves via a “cut and paste” mechanism [1, 6]. On the other hand, retrotransposons represent the largest class of TEs, approximately 37% of the human genome, and they transpose through an RNA intermediate in a “copy and paste” mechanism [3, 6, 7]. Retrotransposons can be further subdivided into three subgroups, namely, the long terminal repeat (LTR) containing endogenous retroviruses, long interspersed nuclear elements (LINEs), and short interspersed nuclear elements (SINEs) [6]. Notably, DNA transposons and most retrotransposons are no longer functional in mice and humans, owing to the accumulation of genetic mutations across evolutionary time [8].

Originally, TEs were thought to be genetic parasites [9–11]. Specifically, the transposition activity of TEs contributes to DNA rearrangements, deletions, and insertions, thereby threatening the host genome with deleterious disruptions to gene regulatory networks. Unsurprisingly, TEs and their spurious activities have been linked to various mutations and diseases [12–16]. However, this traditional view of TEs as parasitic elements is oversimplified, as seminal work by Barbara McClintock on TE regulation of neighboring genes in maize suggested that TEs could harbor rich sources of regulatory elements suitable for host co-option [17]. Work in later years indicated that selective forces may be acting to domesticate certain TEs for regulatory purposes, catalyzing the evolution of eukaryotic gene regulatory networks [18, 19]. Examples of these include RAG enzymes that are involved in the generation of antibody repertoire [20] and syncytin in placental development [21]. In normal development, a specific TE, LINE1, is expressed in neuronal progenitor cells and contributes to neuronal diversity [22]. Other studies further supplemented evidence supporting the notion that a significant fraction of TEs is implicated in transcriptional and epigenetic programs involved in development [21–26] and various phenotypes [27–30].

Notably, TEs have diverged so rapidly that even within mammals, their abundance and activities are highly variable [31]. In light of this, it is remarkable to observe a surge of transcriptional activation of TEs, more specifically retrotransposons, during preimplantation development across various mammalian embryos, albeit with differences in timing and class of retrotransposons [31]. In humans [32] and mice [33, 34], the increase in the transcription of species-specific retrotransposons is evident as early as in the zygote and is maintained up till the blastocyst stage. It remains unclear how disparate TE compositions across the mammalian genomes become involved in a highly conserved process. Yet, this conservation suggests that retrotransposons likely play a crucial role in mediating some aspects of preimplantation development. Although most retrotransposons are nonfunctional fossil remnants in the genome, some families, such as LINE1, remain transposition-competent. However, the observed LINE1 retrotransposition activity is disproportionately low, given its high transcript abundance in mouse embryos [35–37]. Thus, the temporal upregulation of TE transcription during early mouse development may exert additional regulatory functions beyond the mere expansion of retrotransposons.

## 2. TE Expression Coincides with ZGA and Totipotency

Maternal-to-zygotic transition, also known as zygotic genome activation (ZGA), is the first major developmental transition after the fertilization of the gametes. During ZGA, maternally inherited transcripts are depleted, and the quiescent zygotic genome becomes transcriptionally active. ZGA occurs in two phases: (1) minor ZGA, characterized by the synthesis of a small set of transcripts from the paternal pronucleus, and (2) major ZGA, during which reprogramming of the gene regulatory networks and expression of stage-specific transcripts peaks [38–40]. This change in gene expression control is coordinated with changes in the cell cycle, chromatin state, and cellular contents. During ZGA, the parental genomes are epigenetically reset: heterochromatin is lost, DNA methylation depletes transiently [41], and histone mobility increases [42]. For an indepth discussion on epigenetic resetting during preimplantation development, please refer to these excellent reviews [43, 44]. Briefly, this reprogramming process gives rise to a more open chromatin architecture and coincides with the establishment of totipotency [42, 45, 46]. Crucially, two epigenetic machineries involved in the silencing of retrotransposons are remodeled during these early stages: DNA methylation and heterochromatic marks, such as H3K9me3. Deficiencies in these silencing mechanisms can lead to transcriptional activation of distinct TEs in preimplantation embryos, as well as in embryonic stem cells (ESCs). For instance, a subclass of retrotransposons, endogenous retroviruses (ERVs), is one of the earliest transcribed sequences in the mouse 2-cell embryos, during which maternal-to-zygotic transition ensues and cellular totipotency is established [47]. The expression of distinct ERV subclasses during various stages of preimplantation was also observed in human embryos [32, 48].

**Figure 2:**
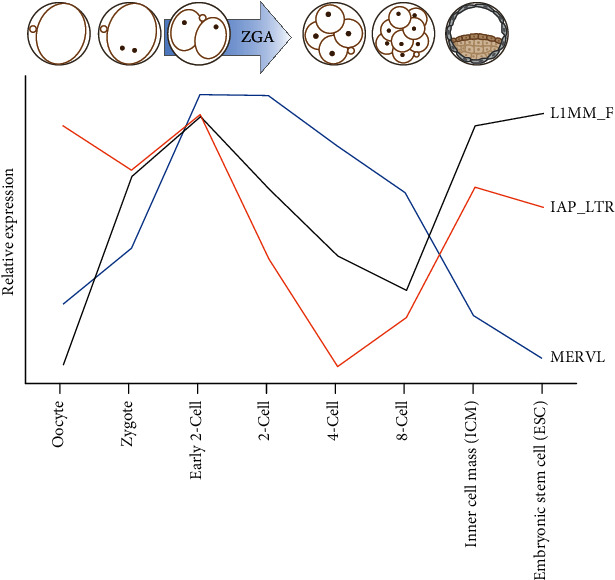
Graphical representation of the dynamics of LINE1, MuERVL, and IAP transcript levels during mouse preimplantation development and in mESCs. The relative expression levels of the TEs plotted are based on the analysis of published RNAseq datasets [45].

Early analyses of gene expression patterns in mouse preimplantation embryos revealed that several retrotransposons have distinct expression levels across different developmental stages (Figure 2). The expression of the earliest characterized retrotransposons include LINE1 and ERVs, namely, class II intracisternal A particle (IAP) and class III murine ERVL with leucine tRNA primer (MuERVL) and MaLRs (internally deleted nonautonomous counterparts of class III ERVL) [33, 49–52]. LINE-1 RNA is detected in the 1-cell zygotic stage embryos, peaks at the 2-cell stage, and remains active throughout embryonic development [33]. IAPs are expressed in oocytes that are rapidly degraded after fertilization and are suppressed during ZGA [53]. Intriguingly, IAPs are reexpressed as development progresses, and their expression peaks at the blastocyst stage [53]. Finally, MuERVL and MaLR expression are restricted to the zygote and 2-cell stage embryos [34, 51, 52, 54].

Efforts to decipher the implications of the retrotransposon expression in the 2-cell context have been facilitated by the discovery of an in vitro cellular model of totipotency, stemming from the work of Macfarlan et al. [54], who identified the existence of a rare subpopulation of mESCs that bear strong molecular and epigenetic resemblance to 2-cell stage embryos, termed 2C-like mESCs. (for a comprehensive review of 2C-like ESC features, refer to [55]). Briefly, both 2-cell embryos and 2C-like mESCs robustly express 2C-specific genes such as DUX [56] and ZSCAN4 [57–59], display high histone mobility [42, 60], and possess relaxed chromatin architectures [45, 56]. 2C-like mESCs also exhibit an increased propensity to contribute to the extra-embryonic lineage, reflective of their expanded cellular potency. Since then, numerous groups have employed the 2C-like system to elucidate molecular factors governing ZGA and uncover molecular features of 2-cell blastomeres [26, 42, 56, 61–83]. Notably, similar to the 2-cell embryos, 2C-like mESCs also exhibit high transcriptional output at LTR elements, specifically at MuERVL elements [54]. Apart from 2C-like mESCs, other ESC models of expanded potency have also been reported; although, they exhibit distinct transcriptomic profiles [84–86]. It is also important to note that the epigenetic and transcriptomic profiles of 2C-like mESCs and 2-cell embryos exhibit differences [87], highlighting nuances between in vivo and in vitro experimental models. Notwithstanding, 2C-like mESCs represent a relevant and tractable model to study ZGA and totipotency outside of the early embryos. Below, we summarize the functional relevance of retrotransposons during development, using studies from both 2C-like mESCs and preimplantation embryos.

## 3. Functional Relevance of Retrotransposons during Preimplantation Development

### 3.1. Retrotransposons Promote TE-Gene Chimeric Transcripts during Preimplantation Development

Throughout evolution, there have been multiple precedents for the co-option of *cis-*regulatory elements of retrotransposons and their role in shaping cell type-specific gene networks. This includes TE contribution to the morphological diversification of the mammalian placenta [88] and regulation of tissue-specific gene expression [89, 90]. LTR elements being utilized as alternative promoters, enhancers, and exons of early embryonic genes have been extensively reported [32, 34, 47, 48, 56]. Indeed, characterizations of mouse oocytes, preimplantation embryos, and 2C-like mESCs revealed that LTRs of MuERVL elements are co-opted as alternative promoters to drive the expression of a high volume of preimplantation-specific genes [34, 91]. These LTR-driven transcripts are termed chimeric transcripts. Similarly, specific primate-specific ERVs are robustly upregulated during each preimplantation stage [32, 48]. Thus, the contribution of retrotransposons, via its *cis*-regulatory elements or transcripts, has emerged as potentially playing a role in the transcriptional regulation of the totipotency program and ZGA.

Notably, it was reported that 90% of mapped chimeric 2C-like transcripts contain Open Reading Frames (ORFs) preceded by LTR elements fused to exons [54]. Moreover, a single-cell transcriptomic analysis of differentially upregulated genes in 2C-like mESCs revealed that a significant proportion contains an MT2_mm (solo LTR of canonical MuERVL) element in close proximity [67], whereas this occurrence is absent in downregulated genes. Therefore, the widespread co-option of LTR-driven transcription could have evolved to coordinate the temporal rewiring of gene networks during ZGA by placing a large proportion of 2C-related genes under the regulation of LTR elements.

### 3.2. LINE1 and MuERVL Transcripts Regulate Gene Expression and Developmental Potency

As previously mentioned, retrotransposons are also rich sources of noncoding regulatory elements. During mouse ZGA, LINE1 RNA performs a function similar to long noncoding RNAs, by recruiting nucleolin/KAP1 to repress 2C-specific transcription factor (TF) DUX and activate rRNA synthesis [26]. The inactivation of the LINE1 expression leads to developmental arrest in 2-cell embryos and promotes transition to the 2C-like state in mESCs [26]. These observations suggest that the *trans*-acting functions of LINE1 are crucial for the modulation of cellular identity during early development.

Overall, there is mounting evidence to suggest that retrotransposons play important roles during development in vivo and cellular plasticity *in vitro*. This is achieved via multiple mechanisms acting in both *cis* and *trans*. Indeed, class III ERVs, which occupy significant proportions of the oocyte and embryonic transcriptomes in both mouse and human, are required for developmental progression [92]. siRNA targeting of 80.5% of the MuERVL elements in mice contributes to the failure of the chimeric transcript expression and a decrease in the GAG protein content [92]. Moreover, even a modest reduction of MuERVL transcripts was sufficient to cause developmental delays, thereby implicating MuERVL in the regulation of early developmental programs [92]. However, it remains unclear whether the resultant phenotype is a direct consequence of the loss of MuERVL transcripts or the depletion of chimeric transcripts. The identification of the exact contributor to developmental abnormalities in MuERVL-depleted cells would be an important next step towards understanding the role of retrotransposons, in particular ERVs, in ZGA and totipotency.

### 3.3. Retrotransposons May Organize Chromatin Architecture in Preimplantation Embryos

The significance of the activation of retrotransposons during ZGA has not been entirely elucidated. In this regard, TE activation could simply be a consequence of increased chromatin accessibility during ZGA, or it could contribute to the unique chromatin features of early embryos. LINE1 and MuERVL have been implicated in modulating chromatin accessibility and organization during early development. The expression of LINE1 in 2C-embryos promotes increased chromatin accessibility, and its subsequent depletion following ZGA is a prerequisite for developmental progression [62, 93]. In a functional study, premature silencing of LINE1 elements led to a decrease in chromatin accessibility, while prolonged activation prevents chromatin compaction and delays developmental progression. Notably, the transcription of LINE1 appears to predominantly impact chromatin structure without overt changes in the global gene expression [62]. These observations imply that LINE1 functions at the chromatin level regulate chromatin accessibility, via its transcriptional activation, and may contribute to the shaping of the early embryonic chromatin architecture in vivo. It is also interesting to note that while LINE1-overexpressing embryos showed developmental arrest, experimental induction of LINE1 chromatin decondensation with an acidic peptide resulted in a milder developmental phenotype [62], hinting at a potential function of the LINE1 transcript itself, perhaps a feedback loop to reinforce chromatin relaxation, that is yet to be elucidated.

Beyond a transcriptional function, MuERVL elements are also involved in shaping the 3D genome during development, as evidenced by Hi-C analysis of MT2_mm and canonical MuERVL, which revealed the establishment of local and global domain boundaries in both 2C-like mESCs and 2C embryo datasets [94], preprint). These domain boundaries are correlated with the transcriptional upregulation of genes downstream of these retrotransposons and increased chromatin accessibility. The potential role of MuERVL in shaping chromatin structure is undoubtedly interesting, but this observation is preliminary and pending further review [94], preprint). While these studies report the involvement of MuERVL and LINE1 in 3D genome organization, their significance in development remains unclear. In line with findings of LINE1-mediated chromatin accessibility, Kruse et al. [94], preprint) also demonstrated that MuERVL-driven domain organization is not related to its gene regulatory activity. Rather, this organization is likely driven by DUX binding and precedes the activation of gene expression from MuERVL elements within the domains. The purpose of these domain boundaries could be twofold: First, during the onset of ZGA, this could concentrate 2C regulatory factors, such as DUX, to promote transcription efficiency. Second, following ZGA, MuERVL-driven transcripts within these domains could be easily packaged into heterochromatic structures to facilitate developmental progression.

## 4. Regulation of Transposable Element Expression during Early Development and ZGA

In the following section, we will outline the TFs and epigenetic mechanisms involved in the regulation of retrotransposons in the context of 2-cell embryos and 2C-like mESCs (Table 1).

### 4.1. Epigenetic-Based Regulation

#### 4.1.1. Histone Modifications

Constitutively repressive H3K9 histone methylation is required for the maintenance of the TE repression following preimplantation development [95] and in somatic cells [96, 97]. In mammals, there are numerous H3K9 histone lysine methyltransferases (KMTs), including Suv39h1, Suv39h2, G9a/GLP, and SETDB1. The depletion of some of these KMTs in mESCs has been shown to promote the activation of specific TEs and transition into the 2C-like state [98, 99]. Of the retrotransposons studied, IAPs are most robustly repressed via the SETDB1-TRIM28/KAP1 silencing complex [100]. Further compaction of IAP into heterochromatin is promoted by the H3K9me3-dependent recruitment of heterochromatin protein 1 (HP1) transcriptional repressor [98, 101, 102]. However, SETDB1-deficient mESCs do not exhibit strong upregulation of MuERVL, indicating that SETDB1-mediated H3K9me3 is likely not responsible for the silencing of MuERVL elements in mESCs. Instead, MuERVL elements are enriched for G9a-dependent H3K9me2 [47], and catalytically active G9a is required for silencing MuERVL LTR-driven transcripts in mESCs [98]. Moreover, G9a depletion in mESCs led to the upregulation of LTR-driven transcripts and a subset of 2C genes [54, 98]. In the same vein, the genomic depletion of SETDB1 in oocytes correlates with the ectopic reactivation of several TEs including IAP and LINE1, but not MuERVL [103]. Intriguingly, H3K9me3 is less enriched on MuERVL elements in 2-cell embryos, suggesting that SETDB1 may have an indirect role in repressing MuERVL elements [104]. Post-ZGA, MuERVL- and LTR-containing retrotransposons are then marked with H3K9me3 from the 4-cell stage onwards, and the H3 histone chaperone, CAF1, is crucial for this H3K9me3-mediated LTR silencing [104].

Krüppel-associated box zinc finger proteins (KRAB-ZFPs) are crucial in mediating TE silencing [101, 105] via its KRAB domain, which contains specific DNA-binding regions and interacts with epigenetic modifiers. This ability to bind at specific genomic sites enables KRAB-ZFPs to direct sequence-specific epigenetic silencing. Epigenetic modifiers such as KMTs (SETDB1), TRIM28/KAP1 scaffold, DNMTs, HP1, and nucleosome remodelers, KDM1A/LSD1, and histone deacetylation (NuRD) complex can then be specifically targeted to TE sites [106–111]. Notably, the role of ZFP-TRIM28/KAP1 interaction in regulating development and pluripotency has been well established [101, 112, 113]. In mESCs, SETDB1-TRIM28/KAP1 is recruited by ZFP809 for retrotransposon repression [114, 115]. The expression of ZFP809 is particularly interesting because *ZFP809* and its MT2_mm initiated chimeric transcripts are both robustly expressed in 2C-like mESCs [45, 114, 116]. This suggests that the MT2_mm expression in 2C-like mESCs could potentiate its own repression by promoting the expression of repressive factors, such as ZFP809, thereby creating an autofeedback loop. Another ZFP is RYBP, which is found to be crucial in the repression of retrotransposons and 2C-specific genes in mESCs, implicating RYBP in the exit from the ZGA program [74].

One of the earliest identified roadblocks to 2C-like reprogramming is the histone H3K4/K9 demethylase, KDM1A/LSD1. KDM1A/LSD1 mutant mESCs harbor significantly higher MuERVL transcript levels [47], indicating that repressive heterochromatin restrains MuERVL transcription [54, 117]. Interestingly, KDM1A/LSD1 is a maternally inherited factor, and its depletion leads to lethality in embryos prior to gastrulation [118]. Furthermore, a requirement of KDM1A/LSD1-mediated chromatin compaction for the exit from ZGA was demonstrated in KDM1A/LSD1 knockout (KO) zygotes that showed developmental arrest at the 2-cell stage [117]. Notably, KDM1A/LSD1 KO zygotes displayed robust upregulation of LINE1 transcripts, but not upregulation of MuERVL [117]. This is in direct contrast to KDM1A/LSD1 mutant mESCs, suggesting that multiple epigenetic regulators likely act in concert to activate MuERVL during ZGA in vivo.

Recently, another histone chaperone, FACT, which mediates H2A/H2B exchange, has also been implicated in the pluripotency-to-2C transition. Specifically, FACT recruits the H2B deubiquitinase USP7 to repress MuERVL- and LTR-driven chimeric transcript expression in mESCs [116]. Loss of either FACT or USP7 in mESCs led to robust upregulation of MuERVL and chimeric transcripts driven by MuERVL, concomitant with the expression of 2C-specific genes. Notably, this finding agrees with the siRNA screen performed by Rodriguez-Terrones et al., which identified ubiquitination pathway proteins, including USP7, as major 2C-like reprogramming roadblocks [73].

#### 4.1.2. DNA Modifications

DNA methylation (5mC) is the most abundant epigenetic modification that plays a major role in the silencing of retrotransposons [119, 120]. 5mC exists in two contexts—canonical in CpG dinucleotides (mCG) and noncanonical CH (mCH, where *H* = *A*, *C*, or *T*). 5mC is established by a group of highly conserved DNA methyltransferases (DNMTs), namely, DNMT1, which preferentially methylates hemimethylated CpG dinucleotides to maintain the 5mC landscape, and DNMT3A/B, which perform de novo methylation at unmethylated CpG [121]. 5mC level changes dynamically throughout development. Here, we summarize the 5mC landscape and its changes, including oxidized 5mC, during development, as well as the current knowledge on mCH in development in Box 1.

#### 4.1.3. Dynamic Changes in Levels of 5mC and Its Oxidized Derivatives during Development

During preimplantation development, the developing zygote undergoes two waves of DNA demethylation. Shortly after fertilization, both maternal and paternal genomes are globally demethylated in the zygotes [122]. 5mC levels reach a relatively low level in the preimplantation embryo, which is followed by increased methylation after the onset of implantation [123]. The second wave of demethylation then occurs in primordial germ cells (PGCs) of the postimplantation embryo. In the first wave, the rapid loss of 5mC is mediated by ten-eleven translocation 3 (TET3), an *α*-ketoglutarate dependent methylcytosine dioxygenase, which iteratively oxidizes 5mC to generate 5-hydroxymethylcytosine (5hmC), 5-formylcytosine (5fC), and 5-carboxylcytosine (5caC) [124, 125]. In contrast, 5mC levels of the maternal genome remain largely unchanged, with a less pronounced accumulation of 5hmC [124–126]. Interestingly, DNA demethylation during the first wave is still an area of intense debate, in which both active and passive models have been proposed. In the latter model, DNA demethylation is thought to be achieved through successive rounds of replication-dependent dilution and lack of 5mC maintenance, as the maternally supplied DNMT1 is excluded from the nucleus during replication [124, 127, 128]. In support of this, the deliberate inhibition of DNA replication blocks DNA demethylation independently of TET3 activity [126]. However, a recent study suggested that DNA demethylation can still occur in the absence of replication in the developing zygote through active DNA demethylation [129].

#### 4.1.4. TE Reactivation Coincides with 5mC Loss and Gain of 5hmC

The loss and gain of 5mC and 5hmC correlated with increased chromatin accessibility and expression of retrotransposons such as LINE1 and MuERVL, but not IAPs, during development. The function of 5mC in the silencing retrotransposon expression has been well characterized and is exemplified by the following DNMT1 loss-of-function study: in mouse midgastrulation embryos lacking DNMT1, the normally silenced IAPs become aberrantly reactivated from the loss of 5mC, leading to developmental delays and embryonic lethality [130]. In embryos lacking DNMT3A/B, MuERVL and 2C transcripts were strongly induced, and this activation is correlated with the global loss of 5mC [131]. Interestingly, IAPs were not reactivated in the DNMT3A/B KO embryos. Mechanistically, DNMT3A/B KO embryos were found to possess elevated expression of 2C-specific TFs DUX, DPPA2, and DPPA4, which are presumably activated due to the loss of the 5mC suppression, thereby potentially explaining the transcription of 2C genes [131]. However, there was no observable increase in the MuERVL and 2C-specific TF expression in DNMT1 KO embryos, despite their significant loss of 5mC [131]. These observations suggest that retrotransposons may be differentially regulated by maintenance and de novo establishment of 5mC and additionally points to a possible noncatalytic function of DNMT3A/B unique to embryos that has yet to be established.

Intriguingly, unlike in embryos, the loss of 5mC in DNMT-KO mESCs does not result in the reactivation of retrotransposons. Specifically, DNMT1 ablation [81, 132] and triple KO of DNMT1, DNMT3A, and DNMT3B [81, 133] in mESCs did not significantly upregulate MuERVL transcription, despite significant global DNA hypomethylation. To address this conundrum, Walter et al. examined the dynamics of retrotransposon reactivation in mESCs in response to global 5mC loss, by readapting the cells in serum-free 2i (PD0325901, CHIR99021) media with vitamin C supplementation. The combined chemical treatment resulted in extensive 5mC depletion that led to the reactivation of select retrotransposons (LINE1, IAP, and MuERVL) [81, 134, 135]. However, following this initial reactivation, the retrotransposons were eventually silenced by acquiring repressive histone modifications. It was found that whereas H3K9me2 levels were globally diminished, and H3K9me3 marks remained unchanged. Importantly, retrotransposons rapidly gained H3K27me3 in response to the loss of 5mC-mediated silencing [134], thereby illuminating an “epigenetic switch” in adaptation of 5mC loss to repressive histone pathways (H3K9me3 and H3K27me3) to maintain retrotransposon dynamics.

This adaptation and alteration in epigenetic repressive mechanisms are also observed during preimplantation development. For instance, H3K9me3 is implicated in repressing select retrotransposons in the absence of active 5mC deposition. Specifically, IAPs and some ERVs are marked with H3K9me3, and these retrotransposons resist 5mC loss during ZGA. Mechanistically, it is suggested that 5mC levels at these retrotransposons are maintained by UHRF1, which recognizes H3K9me3 [136–139]. UHRF1, a cofactor of DNMT1, is essential for maintaining 5mC levels on IAPs in preimplantation embryos [139]. This adaptation is further exemplified by the silencing of MuERVL- and LTR-containing retrotransposons post-ZGA, during which MuERVL and LTRs rapidly acquire H3K9me3 and H3K27me3 following ZGA from the 4-cell to the late blastocyst stage [104]. This increase in H3K9/27me3 correlates with MuERVL- and LTR-containing retrotransposon repression during this period in the absence of 5mC, as zygotic DNMTs are only expressed in the late blastocyst. Collectively, these observations in both early embryos and mESCs highlight the importance of H3K9me3- and H3K27me3-mediated chromatin pathways in retrotransposon silencing in the absence of 5mC.

However, the role of TETs and 5hmC in regulating the retrotransposon expression is less straightforward. In TET1/3 KO mouse embryos, 5mC levels at LTR (IAPs) and non-LTR (LINE, SINE) retrotransposons are higher than controls, which correlate with lower expression of these TEs [140]. Genetic ablation of GADD45, a key interactor of TET enzymes and a component of the DNA demethylation machinery, hindered 2C entry in mESCs [76]. GADD45 is an adapter that directs and tethers TETs to genomic loci for DNA demethylation [141]. The loss of GADD45 function negatively impacts DNA demethylation, as the recruitment of TETs and the required accessory cofactors is affected. GADD45 (GADD45a, GADD45b, GADD45g) triple knockout (TKO) mESCs exhibit higher levels of 5mC when compared to controls and consequently impaired expression of prototypic 2C genes. This result corroborates a previous observation of global 5mC loss during 2C state cycling in mESCs [81]. GADD45 double knockout (DKO) embryos are sublethal, showing impaired upregulation of ZGA-associated genes and reduced implantation success [76]. Nonetheless, some LINE1 elements are upregulated in the DKO embryos, suggesting a repressive role of TET enzymes on LINE1. Indeed, TET1/2 has been reported to repress LINE1 in mESCs [142]. Interestingly, even though TET1/2 and 5hmC are enriched at young LINE1 elements, this DNA demethylation did not result in the reactivation of LINE1, as TET1 was found to recruit the SIN3A corepressive complex to maintain LINE1 silencing in the absence of 5mC [142].

In addition to LINE1, TET2 has also been found to repress MuERVL in mESCs [83]. Mechanistically, the RNA-binding protein paraspeckle component 1 (PSPC1) recruits TET2 to posttranscriptionally destabilize MuERVL and MuERVL-driven RNAs through 5hmC modifications [83]. PSPC1-TET2 can also recruit histone deacetylase 1 and 2 (HDAC1/2) to repress MuERVL transcription. Loss of PSPC1 not only drives the expression of MuERVL but also a subset of 2C-like genes. Notably, this effect of PSPC1-TET2 regulation is specific to TE classes. For example, PSPC1-TET2 interaction transcriptionally activates the class II ERVK (IAP and MusD) expression, but not class II MuERVL in mESCs. Unlike TET3, the TET2 expression is low in 2-cell embryos and only increases during the blastocyst stage [143]. As such, it would be interesting to dissect the different roles of each TET member and further investigate whether PSPC1-mediated TET2 hydroxymethylation could be involved in modulating ZGA exit in vivo. In summary, TETs and 5hmC may potentially exert dual roles—first to relieve 5mC repression during ZGA and second to repress retrotransposon expression as development progresses to later stages.

#### 4.1.5. Histone Chaperones

2C-like mESCs and 2-cell embryos are known to display higher chromatin mobility [42]. A crucial roadblock to 2C-like reprogramming in mESCs is CAF1, a replication-coupled H3/H4 histone chaperone [66]. In 2-cell embryos, it was reported that the p60 subunit of CAF1 is transiently depleted from the replicating chromatin in the early S phase, indicating a decoupling of chromatin assembly with replication during ZGA. This correlates with ATAC-seq observations of large stretches of highly accessible chromatin regions, including MuERVL, in the early 2-cell embryos [45]. This delayed chromatin assembly may transiently render the chromatin more accessible, thereby promoting TE and other 2C gene expressions. Additionally, CAF1 is also responsible for mediating the deposition of repressive H3K9me3 on LTRs, thereby protecting preimplantation embryos from endogenous retrotransposon expression post-ZGA [104]. Interestingly, the replication-associated factor, RIF1, has also been identified to negatively regulate the MuERVL expression in both mESCs and hESCs, and it inhibits mESC transition to the 2C-like state [160]. RIF1 recruits histone modifiers and promotes the establishment of repressive histone marks and DNA methylation, possibly via its interaction with KMTs [160] and CAF1 [161]. Furthermore, the transcriptomic profiles of CAF1 KD and RIF1 KD 2C-like mESCs are highly similar, suggesting that both factors could function in the same axis [160].

#### 4.1.6. Noncoding RNAs

TEs are rich sources of *trans*-acting factors. Notably, TE-derived sequences are highly overrepresented in vertebrate noncoding RNAs, including lncRNAs, siRNAs, piRNAs, and microRNAs [162]. For example, LINE1 RNA acts as a scaffold to recruit the RNA-binding proteins nucleolin and KAP1 and together regulate the exit of the 2C-like state in mESCs [26]. The importance of LINE1 RNA functioning as a scaffold in regulating the 2C-like state and 2-cell embryos is further highlighted by two recent studies demonstrating that N^6^-adenosine methyltransferase (METTL3) and YT521-B homology domain C1 (YTHDC1) m^6^A mRNA reader modulate 2C-like transitions in mESCs [72]. Mechanistically, YTHDC1 binds to m^6^A-modified LINE1 transcripts and facilitates the recruitment of nucleolin and KAP1 to the LINE1 scaffold [72, 163]. Loss of either YTHDC1 or METTL3 results in a depletion of H3K9me3 on the gene bodies of retrotransposons and robust activation of the 2C-like program, including MuERVL [72, 163]. Moreover, YTHDC1 KO embryos displayed developmental defects, reinforcing the importance of YTHDC1-LINE1-nucleolin-KAP1 in finetuning the transcriptional activity of 2C genes and retrotransposons during early development. This finding also hints at another instance of retrotransposons functioning to regulate their own expression via a feedback loop, wherein LINE1 RNA-bound YTHDC1 is specifically recruited to TE gene bodies. Altogether, a diverse cast of epigenetic regulators contributes to the enforcement of specific and timely TE activity.

### 4.2. Transcriptional Regulation of Retrotransposons

#### 4.2.1. DUX Pioneer Factor Directly Activates MuERVL Transcription

In mouse, one of the key TFs involved in the activation of MuERVL is DUX [56, 63, 164], a double homeodomain TF conserved amongst mammals [165]. DUX (DUX4 in human) was first identified to be aberrantly expressed in facioscapulohumeral muscular dystrophy (FSHD) in humans, a disorder that is characterized by an unusually high transcriptional output of ERVs [166]. In mESCs, the ectopic expression of DUX results in the transcriptional activation of MuERVL LTRs and a subset of the ZGA transcriptome corresponding to the 2-cell stage embryo [56, 63, 164]. *In vivo*, DUX was also observed to be upregulated in the early 2-cell embryos, positioning it as a pioneer factor for ZGA [56, 63]. DUX regulates the expression of MuERVL via its interaction with DUX recognition motifs present on LTRs. In fact, a significant proportion of genes expressed during ZGA is in close proximity to LTRs bearing DUX binding motifs. In parallel, the DUX4 overexpression in hESCs triggers the expression of HERVL and a subset of ZGA genes that are expressed in 4-cell human embryos [56]. Throughout evolution, there have been precedents of convergent co-option of TEs as regulatory regions for specific gene networks that define specific cellular states [167]. Therefore, the coevolution of DUX and MuERVL may serve as a means to coordinate ZGA, a complex process governed by multiple genes, with TEs serving as alternative promoters that can only be activated during ZGA by the 2-cell stage specific DUX TF.

A notable target of DUX is the microRNA, miR-344. DUX binds to miR-344's promoter to activate its expression, which then posttranscriptionally represses ZMYM2, a recruiter and stabilizer of KDM1A/LSD1 [71]. The overexpression of miR-344 alone is sufficient to induce 2C-gene and MuERVL expression, indicating that miR-344 is a robust activator of the 2C-like state downstream of DUX. Importantly, transient siRNA knockdown of ZMYM2 in zygotes leads to developmental arrest at the 2-cell stage and more robust MuERVL expression, implicating the DUX-miR-344-ZMYM2-KDM1A/LSD1 axis in regulating the TE expression during ZGA.

#### 4.2.2. Multiple Maternally Inherited Factors Promote MuERVL and LINE1 Transcription

Although DUX is a key inducer of the 2C gene expression program, the DUX expression only begins during minor ZGA [165]. This suggests that upstream maternal factors may be involved in the activation of ZGA, either in a DUX-dependent or DUX-independent manner. Indeed, high expression levels of maternally inherited TFs, DPPA2, and DPPA4 are observed in 2-cell embryos and 2C-like mESCs [77–79, 168]. The overexpression of DPPA2 and DPPA4 induces 2C-like transitions in mESCs, as well as the expression of MuERVL and LINE1 transcripts through the transcriptional activation of DUX [77]. Importantly, depletion of DPPA2 and DPPA4 significantly reduced the efficiency of 2C-like induction in mESCs [77, 78].

In addition to promoting DUX activation, DPPA2 and DPPA4 are implicated in shaping the epigenetic landscape of LINE1 elements that harbor DPPA2-binding sites at their 5′ ends [169]. Mechanistically, DPPA2 and DPPA4 prevent de novo DNA methylation at LINE1 elements so that they remain competent for reactivation during lineage specification [169]. As previously discussed, LINE1 promotes chromatin relaxation during ZGA; in this case, DPPA2 and DPPA4 upregulation during ZGA may also contribute to LINE1-mediated chromatin accessibility.

What regulates DPPA2 and DPPA4? DPPA2 is regulated posttranslationally by the sumo ligase PIAS4, which sumoylates and inactivates DPPA2. Accordingly, PIAS4 is downregulated in 2-cell embryos, during which DPPA2 is active and MuERVL is robustly transcribed [79]. Notably, in a proteomic screen, SUMO2/3 was also found to be involved in DPPA2 and DPPA4 inactivation and impediment of reprogramming to the 2C-like state [170]. The ectopic overexpression of PIAS4 in zygotes impaired the activation of the ZGA program and 2-cell specific genes, including MuERVL, suggesting that PIAS4 inhibition of DPPA2 indirectly regulates the MuERVL expression. This finding also indicates that the SUMO pathway may be implicated in the modulation of 2C-like transition and ZGA. In support of this, sumoylation of PRC1.6 components contributes to the repression of DUX, a potent activator of the MuERVL expression [171]. Furthermore, the SUMO pathway has also emerged as a repressor of MuERVL in mESCs through a genome-wide siRNA screen for proviral repressors [161].

Apart from DPPA2/4, the mammalian-specific factor STELLA (encoded by *Dppa3*) is also maternally inherited and is required for proper preimplantation development of mouse embryos [92, 172, 173]. Early studies have implicated STELLA in the protection of the maternal pronucleus from TET3-mediated active demethylation in oocytes and maintenance of DNA methylation at a subset of imprinted genes and retrotransposons [174, 175]. However, the dispensability of TET3 and maintenance of imprinted genes for preimplantation development indicate that STELLA could play additional roles in preimplantation development [176, 177]. Indeed, Huang et al. demonstrated that STELLA maternal/zygotic knockout (M/Z KO) 2-cell embryos showed impairment in ZGA and a failure to upregulate 2-cell, LTR-driven, and MuERVL transcripts [92]. The aberrant MuERVL expression is directly attributed to the loss of STELLA, given that MuERVL can be activated in arrested 2-cell embryos with functional STELLA [117]. Interestingly, the overexpression or loss of *Dppa3* did not significantly alter the MuERVL expression levels in mESCs, highlighting context-specific differences.

In type 2 FSHD (FSHD2), the DUX4 overexpression is most often due to loss-of-function of the structural maintenance of chromosomes hinge domain 1 (SMCHD1) gene [178]. In mice, SMCHD1 is also responsible for the DUX repression following ZGA. SMCHD1 mRNA transcript and protein are maternally inherited, and transient depletion of SMCHD1 mRNA in zygotes leads to the protracted DUX expression and developmental defects [179]. SMCHD1 KO mESCs also display upregulation of DUX and MuERVL [70]. Mechanistically, SMCHD1 sequesters TET proteins from DUX promoter, leading to hypermethylation and silencing of DUX. Recently, our lab also identified a maternal factor, negative elongation factor A (NELFA), which partners with DNA topoisomerase 2A (TOP2A) to promote 2C genes and TE expression in mESCs. We determined that the NELFA overexpression in mESCs is sufficient to activate the 2C program, including robust DUX and MuERVL activation [65]. Consistent with our observations, a role of TOP2A-mediated DNA double-strand breaks was implicated in ZGA in the *C.elegans* germline [180].

Several evidence also point to a role of the DNA damage response in ZGA/2C induction [59, 61, 181–183]. Notably, in a recent study by Grow et al. [64], it was found that p53, a key transcriptional effector of DNA damage, mediates the expression of DUX and 2C-specific transcripts, including MuERVL in mESCs [64]. Crucially, p53 is maternally inherited, and its activation coincides with the accumulation of endogenous DNA damage in the early embryos. Similar to the observed requirement of p53 to activate DUX and 2C transcript expression in mESCs, p53 maternal/zygotic KO embryos showed lower, but not complete elimination of DUX and 2C gene expression levels. Importantly, DUX4 in human iPSCs is also activated by p53, suggesting that p53 could play similar regulatory roles during human ZGA.

#### 4.2.3. GATA2-miR-34a Axis Regulates the MuERVL Expression

GATA2 is another TF implicated in regulating the 2-cell gene network in mESCs and is under the regulation of the microRNA, miR-34a [80]. Loss of miR-34a resulted in the upregulation of MuERVL elements. Investigation of the 18 most highly upregulated MuERVL loci revealed the presence of GATA2 TF binding sites [80]. Interestingly, not only is GATA2 upregulated in 2-cell embryos but its expression pattern is also correlated to the MuERVL expression during preimplantation development, thereby implicating GATA2 as a transcriptional activator of MuERVL in 2-cell embryos. Indeed, GATA2 can bind to MuERVL LTRs when overexpressed in miR-34a KO mESCs, and loss of GATA2 led to the impaired MuERVL expression in these cells. However, the overexpression of GATA2 alone in wildtype mESCs is not sufficient to induce the MuERVL expression, suggesting that other miR-34a targets in addition to GATA2 may be required to cooperatively bind and activate the MuERVL expression. Another notable observation is that, similar to what was observed in DUX KO mice, miR-34a KO embryos can undergo successful preimplantation development [80], reinforcing the notion that MuERVL regulation during ZGA is modulated by complex and partially redundant regulatory networks.

#### 4.2.4. ZSCAN4 Is Both a TF and Scaffold for Chromatin Modifiers at MuERVL Sites

ZSCAN4 is another 2C-specific TF that is robustly expressed in both late 2-cell embryos and 2C-like cells. It consists of several paralogs (ZSCAN4a-f and three pseudogenes ZSCAN4-ps1-3) [58]. ZSCAN4 functions to maintain telomere length and ensure genomic integrity in mESCs [59, 184, 185]. This gene cluster has also been implicated in promoting 2C-like transition and activating preimplantation genes [57, 59, 81, 186]. Moreover, ZSCAN4-depleted embryos display developmental delays during ZGA [58]. In agreement with these observations, ZSCAN4c, in particular, directly binds to and activates the enhancer of MT2_mm and increases the 2C-specific gene expression in mESCs [187]. Mechanistically, ZSCAN4c recruits BAF-containing chromatin remodeling complex, GBAF, via its SCAN domain, to MT2_mm sites [187]. It has been proposed that ZSCAN4c-GBAF complex could be responsible for the deposition of activating histone marks (H3K27ac, H3K4me1, and H3K14ac) on MT2_mm, highlighting an epigenetic function of ZSCAN4c in regulating the TE expression. Given that the expression level of ZSCAN4c is significantly higher in 2-cell embryos compared to mESCs, the authors speculated that ZSCAN4c could similarly activate MT2_mm during ZGA. Additionally, it was observed that DPPA2, DPPA4, and DUX were highly upregulated upon the ZSCAN4c overexpression in mESCs. However, unlike the DUX overexpression, the overexpression of ZSCAN4c in DPPA2/DPPA4 double KO mESCs could not promote 2C-like transitions [77]. The upregulation of DUX is interesting given that DUX is known to bind and activate the ZSCAN4 cluster in 2C-like mESCs [56]. This implies that DUX may not be the sole driver of the 2-cell program, and that 2-cell specific TFs may also function to reinforce each other's expression, thereby contributing to a positive feedback loop to activate the 2-cell gene expression program.

#### 4.2.5. CCCTC-Binding Factor (CTCF) Restrains 2C-Like Reprogramming

The chromatin architecture protein, CCCTC-binding factor (CTCF), was recently discovered to be a barrier to 2C-like reprogramming [73]. CTCF is a zinc finger binding protein with roles in chromatin compaction and the insulation of topologically associated domains (TADs) [188]. Loss of CTCF promotes entry into the 2C-like state, in a ZSCAN4-dependent manner [73]. Interestingly, this study found that the upregulation of ZSCAN4 precedes that of DUX and MuERVL, placing ZSCAN4 expression upstream of DUX. Consistent with a role of CTCF in restraining 2C gene induction, the CTCF expression is lower in 2-cell embryos than in the ICM, the former characterized by a more relaxed chromatin state that is associated with weak TAD boundaries [189]. Taken together, CTCF may be a major repressor of the 2C-like state in mESCs, as well as during ZGA *in vivo*.

## 5. Conclusion

The contribution of TEs to gene regulation and chromatin dynamics is evident in the functional conservation of specific TE subclasses, even though there is limited conservation of TE sequences and activity of subclass type across species, and extensive TE polymorphisms are prevalent within species [190–192]. In particular, the convergence of retrotransposon regulation illustrates the importance of its precise expression during development. It is now clear that the reactivation of specific retrotransposons during early embryogenesis is not merely a consequence of genome-wide reprogramming, but exerts key biological functions. In fact, MuERVL activation alone, without DUX induction, is sufficient to induce the expression of a subset of 2C genes, reinforcing that the critical regulatory role MuERVL plays during early development [83]. Nonetheless, how different retrotransposons participate in sculpting the totipotency program and their mechanisms of action remains open questions.

Recent studies have highlighted key differences between retrotransposon expression and regulation in 2-cell embryos versus 2C-like mESC counterparts. For example, DUX is a key driver of mESCs to 2C-like transitions [54], but is not essential for ZGA in vivo [193], and its expression only activates a subset of the 2-cell program [56, 63, 164]. Moreover, DUX only occupies a quarter of the accessible chromatin in 2C-like cells, suggesting that this system may not fully recapitulate the complexity of ZGA [56]. Indeed, maternal and zygotic DUX KO mice are able to develop into adulthood, and a subset of presumably DUX-activated 2C genes in 2C-like mESCs can still be activated in DUX KO mice [78, 194]. Therefore, these findings suggest that multiple TFs that remain to be determined are likely involved in the regulation of the 2-cell transcriptional program.

These emerging studies on the dispensability of factors hypothesized to play key roles in the activation of retrotransposons and 2C genes during embryonic development are indicative of overlaps between the functional roles of retrotransposons and the pathways regulating their transcriptional activities. In this regard, it will be important to assess whether findings from 2C-like systems can be recapitulated in early embryos. It is likely that the transcriptional output of retrotransposons is dependent on the contribution of both nuclear factors and chromatin dynamics. Taken together, these observations paint a highly complex landscape of retrotransposon regulation in totipotency, delineating how specific classes of retrotransposons function and their dynamic nature of regulation will be integral in illuminating the diverse roles of retrotransposons during early development and cell fate determination.

## Figures and Tables

**Box 1:**
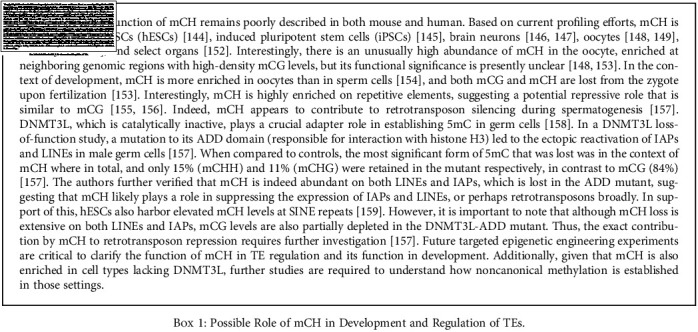
Possible Role of mCH in Development and Regulation of TEs.

**Table 1:** Factors with roles in the regulation of the retrotransposon expression during (1) mouse ZGA and (2) in 2C-like mESCs. Not all factors demonstrated to be involved in retrotransposon regulation in ZGA are involved in the context of 2C-like mESCs, and vice versa.

	ZGA	2C-like mESCs
*Transcription factors*		
DUX	[63]	[56, 63, 64, 164]
p53	-	[64]
DPPA2/DPPA4	-	[77, 78]
NELFA	-	[65]
GATA2	[80]	[80]
ZSCAN4	-	[57, 59, 77, 186]

*Posttranscriptional regulators*		
miR-344	-	[71]
miR-34a	-	[80]

*Posttranslational modifiers*		
SUMO2	-	[161]
PIAS4	[79]	[79]

*Chromatin-associated regulators*		
KMTs (Suv39h1, Suv39h2, G9a/GLP, SETDB1)	[104]	[98, 99]
KDM1A/LSD1	[117]	[47]
ZMYM2	[71]	[71]
FACT complex	-	[116]
USP7	-	[67, 116]
DNMT1-UHRF1	[139]	-
TET2-PSPC1	-	[83]
CAF1	-	[66]
GBAF	-	[187]
STELLA	[92]	-
SMCHD1	[179]	[70]
LINE1	[26, 163]	[26, 72, 163]
ZFPs (ZFP809, RYBP, REX1)	[115]	[74, 114, 195]
HP1	-	[98]
TRIM28/KAP1	-	[98, 101]
RIF1	-	[160]
